# Macro level system mapping of the provision of mental health services to young people living in a conflict context in Colombia

**DOI:** 10.1186/s12913-024-10602-2

**Published:** 2024-01-25

**Authors:** Sarah-Jane Fenton, Juan Roberto Rengifo Gutiérrez, Monica Pinilla-Roncancio, German Casas, Francy Carranza, Sanne Weber, Paul Jackson, Juan Pablo Aranguren Romero

**Affiliations:** 1 University of Birmingham, Birmingham, UK; 2 Universidad de Los Andes, Bogota, Colombia

**Keywords:** Health; Mental Health Policy, Childhood and youth population, Colombia, Internal Armed Conflict

## Abstract

Colombia has one of the longest running internal armed conflicts, which has significantly impacted the mental health of the population. This article is the first to present a national level mapping of the provision of mental health services to *young people* living in Colombia, through detailed review of documentation, interviews with key stakeholders and quantitative analysis of existing data on mental health and suicide. It explores the existing public mental health provision in the country, focussing on where mental health resources are concentrated and how these are implemented. We use this mapping to understand how the current mental health system in Colombia fits with international approaches to youth mental health. We show that whilst mental health policy is variously framed (biomedical, biosocial, psychologically or through human rights), Colombian policy clearly focusses on a differential approach. This differential approach shapes service provision to target support at those in need, consequently neglecting whole population level mental health support. This means that not all stakeholders were clearly articulated or included in policy and that key institutional stakeholders, such as the education sector, were not linked to implementation plans or activity. Policy approaches were also over-centralised with little cross-institutional collaboration. Youth were specifically missing from services, as was explicit understanding of the intergenerational effects and impact of conflict. This was exacerbated by unequal distribution of mental health care services concentrated in populous, urban areas away from conflict-affected regions. Suicide is the second most prevalent cause of death with 10% of population who were recorded as dying by violence, dying from completed suicide. Triangulation implies a strong relationship between suicide and poorer access to professional support in conflict-affected areas and suggests that international frameworks and policy approaches to supporting youth mental health have been insufficiently adapted for conflict and post conflict contexts.

**Supplementary Information:**

The online version contains supplementary material available at 10.1186/s12913-024-10602-2.

## Introduction

Mental illness is cited as a key threat to health, wellbeing and productivity and the successful transition into adulthood, having now become the leading cause of disability and poor life outcomes for young people [1]. For young people living in a conflict or post-conflict context, there is increased likelihood of having poor mental health [2]. It is therefore essential to understand how best to support youth mental health and promote health equity [3] in conflict and post-conflict contexts.

The 2020 *Global Framework for Youth Mental Health* highlighted how mental health disorders presented a significant portion of the global disease burden (35%), amounting to more economic losses than cancer and diabetes combined (22%) [1]. The epidemiological evidence underscored the importance of developing robust supportive systems for mental health [1]. The report emphasised this in relation to young people from Low- and Middle-Income Countries (LMICs), arguing that 75% of all mental disorders onset before the age of 25, and nine of ten young people live in LMICs [1]. In response to this challenge, a framework was developed, which calls on governments internationally to adopt “eight principles, underpinned by a series of practices, to guide local implementation of youth mental healthcare” [1, p. 11]. These principles included:

1. Rapid, easy and affordable access.
2. Youth-specific care.
3. Awareness, engagement and integration.
4. Early intervention.
5. Youth partnership.
6. Family engagement and support.
7. Continuous improvement.
8. Prevention.

We need to understand how youth mental health provision currently operates in conflict settings, such as Colombia, in relation to these internationally developed principles, to determine if these goals are feasible, and to decide how to prioritise these appropriately.

Understanding how to support youth mental health in conflict and post conflict contexts is critical as the prevalence of mental health and psychosocial problems in post-conflict and humanitarian settings is high [4]. There may also be significant disruption to the architecture of formal health services including mental health, thereby creating equity of access issues leading to conflict becoming a key social determinant for health [3]. Any systemic impacts have the potential to exacerbate individual effects through disrupted or lack of provision of services and resources. For children and young people who have less agency than adults due to their developmental stage, any health inequity is magnified.

The impact of armed conflict on children and young people’s mental health has been shown by Betancourt et al. [5, 6] to increase mental health problems related to symptoms of post-traumatic stress disorder (PTSD), anxiety disorders and depression among children disengaged from war [7]. Direct experience of war can operate as a determinant of poor mental health requiring clinical involvement, but different psychosocial factors in the post-conflict period also contribute to the risk of developing mental disorders [6].

This article presents a national (macro) level system mapping of the provision of mental health services to young people (0–25 years) living in Colombia. The mapping explores the existing mental health policy and provision services for young people in Colombia. We use this mapping to understand how the current mental health system fits with the WEF [1] framework; what the existing barriers and facilitators of policy implementation are; and how this mapping helps us to understand or integrate new perspectives/approaches to the delivery of mental health services for young people in conflict or post-conflict contexts.

## Context

Colombia has one of the longest internal armed conflicts [8] with one of the highest number of victims (internally displaced persons, killed, disappeared) in the world [7]. A consequence of the violent fifty year conflict between the military, paramilitary and guerrilla groups has been a significant impact on mental health in general [9]. The 2016 Final Peace Agreement between the Colombian Government and Las Fuerzas Armadas Revolucionarias de Colombia—Ejército del Pueblo (FARC-EP) represented a chance to create comprehensive reform, democratic openness, political inclusion, and a solution to the illicit drug problem. Despite the Final Peace Agreement (2016), ongoing territorial displays of violence fuelled by economic inequality and activities such as drug trafficking and illegal mining, have fractured the scope of peace as a process in the regions and perpetuated forms of oppression against the civilian population in peripheral, non-centralised and mostly rural areas of the Colombian national state [10, 11]. It also recognized the emotional suffering and economic and material damage that the conflict caused to the Colombian victims and population. Research into the armed conflict on the Pacific Coast demonstrates increasing institutional weakness and consequent impacts on equity of access to key services [12, 13].

The experience of armed conflict (either direct or indirect) is pervasive in children and young people’s lives in the Colombian context. Few studies exist that focus solely on the effects of armed conflict in Colombia on young people [14], and fewer still that focus on their mental health [15, 16]. According to the National Mental Health Survey (NMHS) [17] 4,7% of Colombian children and adolescents have at least one mental health disorder, and this prevalence increases for those young people living in conflict areas and among victims of violence.

There are multiple direct impacts on the lives of children and young people including through the recruitment into participating in violence [18]; sexual abuse of women and children [18]; loss of family members, home, or access to education through internal displacement [19] as well as risk of death or serious injury. The Truth Commission that was established as a result of the 2016 Peace Agreement focusing on victims’ conflict experiences, reported that 63.5% of the testimonies heard from children and adolescents reported mental health problems including insomnia, migraines, depression, anxiety, recurrent involuntary memories, and suicidal ideation [17].

The armed conflict also has indirect effects on wider groups of children and young people, with the Colombian NMHS reporting on displaced adolescents that 5.3% migrated due to violence [17]. Of those 19.8% experience suicidal thoughts compared to 5.8% of non-displaced adolescents, and 9.1% have attempted suicide compared to 2.1% of non-displaced adolescents. Delivery of services to wider groups of children and young people is therefore a critical policy priority, and establishing a feasible evidence-based framework for achieving this is essential.

## Methodology

The wider research project, of which this study forms part, is shaped by ecological systems theory, which offers a framework through which to examine an individual’s development, and the relationships that they have with both the people they have around them in their network and wider society [20]. Through integrating an ecological systems perspective, this mapping of the national (macro level) context is importantly understood as a moderator of regional and local (meso, micro level) contexts in which young people (individual level) are experiencing development within a wider context of conflict.

This research adopted a mixed methodology including: (1) a documentary policy analysis to understand youth mental health provision (phase 1); (2) interviews with key policy makers and stakeholders responsible at the national level for policy delivery and implementation (phase 2); and (3) a quantitative analysis of three key datasets to understand the resources available for youth mental health within Colombia (phase 3) (see Table 1).

**Table 1:** Summary of data collected across the three phases

Phase 1: Documentary policy analysis	Phase 2: Qualitative interviews with key policy stakeholders	Phase 3: Quantitative mapping of key datasets
• 45 documents including critical policy, national plans, and legislation • Of these 27 were primary source documents, 7 secondary source documents, and 13 contextual documents.	• 9 interviews with key (macro level) policy stakeholders • Of these 1 was from health; 3 were from education; 2 were from legal or justice; 3 were linked to Non-Governmental Organisations (NGOs).	• Registro de Proveedores de Salud en Colombia (REPS) for September 2021 • National Mental Health Survey (NMHS) from 2015 • Administrative records on mortality from 2018 and 2019 We analysed the suicide rates of the country.

### Phase 1– documentary policy analysis data collection and extraction

We conducted a documentary analysis of 45 documents including critical policy, national plans, and legislation including 27 primary source documents, 7 secondary source documents, and 13 contextual documents. The documents referenced throughout the results section and included in the analysis are listed in Appendix (A1). Secondary sources were used as references where they were tangentially relevant to the research questions (such as policies for Non-Governmental Organisations hereafter NGOs); contextual documents were not directly relevant to the research questions but provided information to help develop deeper understanding of the wider context (such as legislation linked to system architecture). Documents selected for inclusion were those that directly addressed aspects of child, adolescent or youth mental health and/or service provision.

The primary source documents were imported into NVivo (release 1.5.1) and interpretatively thematically analysed. This interpretative approach looked for the implied assumptions or underlying ideologies about mental health, youth, and conflict enabling overt and explicit data to be extracted that reflects “the rhetoric of policy environment and the government’s intentions”, as well as implicit data or that which “reflects the ideology” [21, p. 259]. A hybrid approach of both deductive and inductive coding during the documentary analysis, which facilitated iterative development of codes, further improved the rigour of the process in linking it back to the research questions [22].

The documentary policy analysis enabled conceptualization of 15 key themes (initially identified through analysis by S-JF, JRR, MVP) based on the research question linked to the WEF criteria, which were reported in three aggregate categories (derived by the whole team through an iterative process at team meetings): contextual descriptions of the system (definitions, causal factors, understandings of conflict, descriptions of public health, health services, mental health promotion, and the policy context); policy reach (recipients by age range, specific groups, policy level, and linkages across sectors); and policy implementation (principles, policy and barriers).

### Phase 2– semi-structured interviews with policy makers and stakeholders at the national level

We carried out nine semi-structured interviews with key informants who were purposively selected given their role in relation to young people and/or mental health policy. The interviews sought to understand the framing of youth mental health in Colombia and the practical and resource constraints in implementing policy in this area. Participants held key national roles related to mental health policy implementation in different contexts, including universities, schools and ethnic and ex-combatant communities. Stakeholders were identified during the documentary analysis as having a responsibility for either informing, building or delivering policy. Transcripts were analysed using qualitative data analysis software NVivo (release 1.5.1). A joint UK-Colombian team used reflexive thematic analysis to avoid a purely deductive (based around the interview questions), or wholly inductive (drawn from the participant narratives) approach to coding and the resulting themes. Cross-comparative analysis across the coding for the policy documents and the interview transcripts took place and where appropriate we coded to similar themes across both data types.

### Phase 3– secondary data analysis of National Register of Health Providers, the National Mental Health Survey and national mortality administrative register

To understand the gaps in the distribution of mental health services and how this relates with a different prevalence of mental health problems or higher suicide rates in Colombia, we used three sources of data. First, we analysed the Register of Health Providers (REPS) from September 2021, which included the official number of registered providers in psychology, psychiatry, and other mental health services in the country. Also, we computed the prevalence of depression, anxiety and other mental health illness for individuals aged 12 to 25 years and the demand of mental health services of adolescents (12 to 17 years) and young adults (18 to 25 years) using the National Mental Health Survey (NMHS) from 2015, which is the only national dataset that has included questions about different mental health illnesses in the country. The NMHS is representative at the national and regional level for persons seven years or older and it uses 12 validated scales to analyse the mental health of individuals in Colombia. The total sample of the survey is 11,665 individuals and 10,123 households. Finally, we computed the mortality as a result of suicide using administrative records on mortality from 2018 to 2019. Mortality administrative registers include the main cause of death, and using this information we calculate the number of people who committed suicide in each year for individuals 12 to 25. Using the results of these analysis, we calculated the correlation between the number of mental health providers and the number of deaths as a result of suicide in 2019 to understand the association between provision and suicide rates, as a proxy of mental health unsatisfied needs.

The results of the phases above were triangulated in line with the underpinning ecological theory and WEF (2020) framework, which inspired the analysis and the emerging codes and themes. This analysis was subsequently synthesised through iterative discussion across the team into three key findings reported in the results: significance of the differential approach in Colombian mental health policy; reach of policy and implications for implementation; and, impact of armed conflict on children and young people’s mental health needs and importance for future policy development.

## Results

### Significance of the differential approach in Colombian mental health policy

**Fig. 1:**
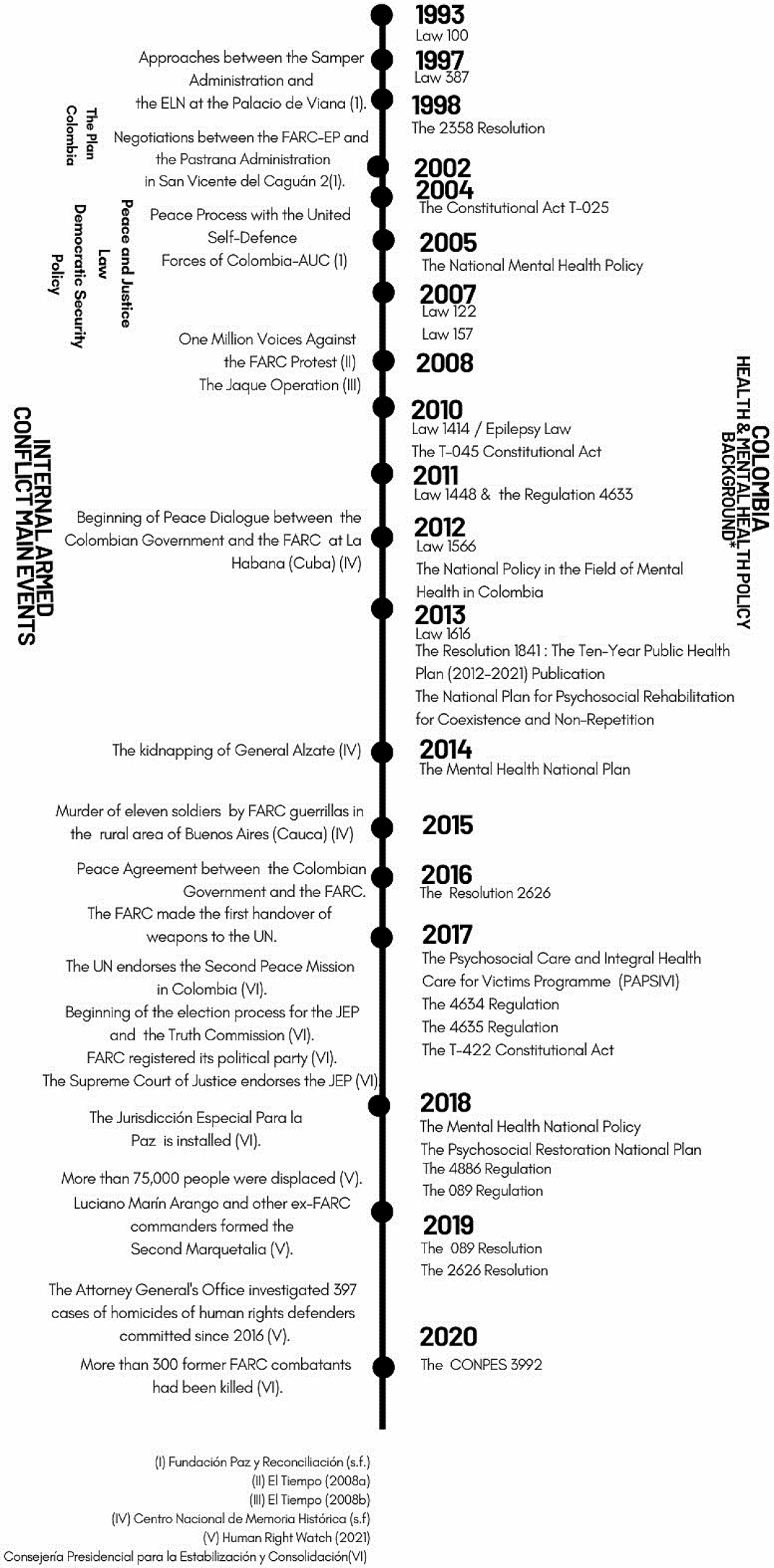
Colombian Mental Health System Timeline (from 1993 to 2020)

Colombian mental health policy has evolved over the past twenty years (see Fig. 1) resulting in a system architecture that enshrines the right to access healthcare and mental health support through legislation (see D3). A clear policy ambition across the documents was to improve the population’s quality of life, and this included improving mental health (D3).

Definitions or the framing of mental health within the documentary policy analysis tended to be biomedical (D1, D5, D8, D12, D14, D16), biopsychosocial (D15, D17, D18, D23, D27), human rights based (D4, D9, D10, D14, D17, D21), or linked to psychological definitions such as wellbeing (D15, D18, D26). However, through the documentary analysis of both policy and legislation, it became clear that the *differential approach* adopted within Colombian policy and the linked desire within policy and legislation to prioritise services for those most vulnerable, particularly as a result of conflict, was integral to understanding the development of mental health systems and services in the Colombian context. The adoption of the differential approach within policy (D14, D15, D18, D24, D26, D27) has been a central guiding principle connected to both biopsychosocial and rights-based framings of mental health. This approach is described in Resolution 2626 (2019, p. 3) as:

*“The differential approach recognises that there are populations with particular characteristics due to their age, gender, ethnicity, condition of disability or victims of violence, among other situations that place them in a situation of disadvantage and greater vulnerability for the exercise of the right to health (…). It implies developing a process of adaptation (…) of the available service structures to the characteristics of the population and the territories, as a critical success factor in the performance of the health system to close the gaps in health outcomes”.*

This understanding shaped policy aims around recovery, rehabilitation and reintegration to reconstruct the “social fabric and the strengthening of trust between the members of the collective and the State” (D23, p.9). Importantly, this differential approach was adopted in relation to children, whereby according to Decree 4633 of 2011 (D10), there must be differential protection measures for children in the context of the armed conflict. Such measures aimed to protect life and physical integrity, including prevention of association with armed actors and groups. This resulted in specific targeted mental health support programmes working with conflict victims (such as The Psychosocial Care and Integral Health Care for Victims Programme hereafter PAPSIVI). Programmes are intended to operate within a framework of comprehensive repatriation and are intended to rehabilitate individuals through mitigating the physical, mental and psychological effects on conflict victims (D10).

The adoption of a differential approach was mirrored not only in policy rhetoric articulating services but also by policy makers:

*“I see it’s necessary to continue developing work that addresses more and better the particularities of population groups, above all, the groups who are most affected by violent situations the country has been experiencing for a long time (E006)”.*

This conclusion was visible in other narratives, where it was felt that the attention to population level or individual suffering was either residual or non-existent (E002, E006, E005, E007), and that the magnitude of the damage caused by conflict was not taken into account when designing health plans (E002).

The documents made explicit reference to the superficiality of international or Western concepts of mental wellbeing for populations when operating in a conflict or post-conflict context. This was coupled with a stated need for evidence alongside political support and integration of mental health policy into broader national policy in order to drive change (D24).

When thinking about the mental health of the whole population rather than targeted interventions focusing on the victims of the conflict, mental health *promotion* received policy attention. Resolution 4886 of 2018 (D24) recommended, following evidence presented in the National Mental Health Survey (2015), that mental health promotion targeted individuals, families, and communities. This was to be achieved through co-responsible sectors using a framework for Comprehensive Care Model (MIAS) and was highlighted within the Ten-Year Public Health Plan (D14). Intersectoral coordination was integral to public health policy and appeared in Resolution 2626 of 2019 (D17), indicating that the Primary Health Care Strategy might allow intersectoral coordination to provide comprehensive and integrated care, from public health, health promotion, disease prevention, and diagnosis. The Primary Health Care Strategy was made up of three integrated and interdependent components, (1) health services; (2) intersectoral/trans-sectoral action for health; and (3) community and citizen social participation. This focus on citizen social participation and the emphasis on individuals, families and communities is a helpful context of policy to consider when thinking about the whole population’s mental health in the Colombian context.

It is also helpful to understand what is seen or described within policy to be contributing to poor mental health. Documents often focused on the social determinants of health, including: psychoactive substance abuse and prevention of suicidal behaviour (D14; D25); rural to urban migration (D2, D3, D4, D10); the dismantling of community support networks (D14, D15, D24, D25, D26, D27); poor access to services (D14); inequality both between groups and between territories and regions (D14, D15); socioeconomic problems and living conditions (D14, D27); gender in relation to discrimination, inequality and power relations (D15, D14, D27); and exploitation, violence and sexual abuse (D10).

Despite these drivers being identified, the documentary and interview analysis indicated that service delivery is still aligned with a differential victim focused approach. Therefore, a possible unintentional policy consequence is there has historically been weak articulation of a general population approach to mental health and a lack of linked implementation plans.

The documentary analysis revealed lack of clarity about sub-populations of interest and missing populations. For example, only one document (D18) directly identified the needs of LGBTQ young people. It was unclear what type of evidence (i.e., epidemiology prevalence or qualitative experience data) supported the prioritisation of specific groups within policy. Whilst there was some mention of Indigenous mental health (D10) there was no prioritisation around ethnic groups.

Other groups did appear specifically mentioned within policy, such as women. Whilst women were identified as being a population of interest as victims, this was not explicitly tied to their identified mental health needs. The dominant way of framing women within policy was as being at risk (D10, D15, D26) in relation to vulnerability (D2, D10, D15, D18, D19, D20) sources here); and as victims (D10, D15, D18, D19, D20, D23). Women were identified as being in need of additional support within health and other public sectors as a result of the impact of conflict in relation to their witnessing or experiencing violence (D10, D15, D18, D19, D20); displacement (D4, D10, D15); sexual violence and sexual health (D10, D15); impact on their role in particular disruption to and impact upon domestic life (D10, D15, D18); in relation to the loss of both socio-economic opportunities and social capital (D10, D15, D18, D27); and in relation to their experience as victims requiring highly complex support including psychological/psychiatric care (D10, D15, D18). Their mental health needs were directly related to their status as victims witnessing or experiencing violence (particularly in relation to the torture/disappearance/recruitment of, spouses, children, other relatives, or themselves). Whilst some documents identified intersectional issues and framed the multiple identities of women formed from social, cultural, and historical issues linked to power structures, the predominant presentation of gender lacked a nuanced understanding of the general mental health of women in Colombia, both those directly and indirectly affected by violence including those who would not constitute ‘victims’(D10).

Interviews revealed that there was an appreciation amongst policy makers that there may be groups who are currently missing from policy and further knowledge is needed to deliver culturally appropriate services for Indigenous Colombians, particularly Indigenous children, and young people; and Afro-Colombians (E002, E008, E009). Participants also recognised that adopting an intersectional approach was increasingly important:

*Mental health disorders in diverse groups of young people (…) So they may be Afro, Indigenous, trans, or gender and sexually diverse, who are in transition of their cultural identities, etc. (…) A public policy should first understand the complexity of mental health, (…) understanding its complexity implies being able to apply an intersectional approach, right?* (E002).

Despite this, one participant interestingly reflected that implementation was the issue:

*I think that, in terms of policies and guidelines, these (…) are more and more sensitive as regards differences, intersectionality. I think the main difficulty still lies in the implementation of these policies and guidelines (E006).*

Stigma and stereotyping were cited as being particular barriers to both the development of mental health support, and engagement in service provision:

*The demand is clearer in the population of victims. The population who is not a victim, I think that still there are many stereotypes and stigmas associated with mental health. We experience this as well in a very clear way with the former combatant population. This is for the ‘crazy’, this is for the ‘weak’, and there everything related to gender dynamics starts to appear, this is for ‘women’ (E008).*

This illustrates that targeting may lead to misinterpretation or unexpected consequences, e.g. ‘mental health services are for victims, who are predominantly women’. This may contribute to problems that might be associated with victim-focused rather than whole-population approaches to mental health. Our research shows that current policy potentially fails to reach diverse groups of young people.

### The narrow reach of policy and implications for implementation

Understanding the *reach* of policy–in terms of who services are for, the populations served by existing services, and identified needs, is critical to understanding how feasible the two first WEF (2020) principles (rapid, easy affordable access and youth specific care) are to implement.

In considering the reach of policy and how this translated into implementation, we examined how age was constructed and what ages were represented within policy. Of the 27 documents analysed, two are applicable across all age groups and discuss the life-course; six explicitly consider children, youth or adolescence; and 19 do not refer to age or are not age specific. Generally, youth, childhood, and adolescence are conflated and the terms used interchangeably. References to children were mostly framed in relation to rights or protection (D4, D10, D14, 15); whereas framings of youth tended to focus more on risk, rights, and inequality (D14, D10, D15, D25). Where an awareness of young people is present, their needs are not explicitly addressed as distinct and there is insufficient understanding of developmental needs. This lack of articulation of youth in policy results in missing links to explicit actions and unclear focus within policy, which weakens the *reach* of policy to these groups and thus the effective targeting of services.

Secondly, our research shows that mental health services are siloed and over-centralised. Whilst the role of mental health promotion and the emphasis on community was evident in policy, what was notably absent was a focus on the role of education settings. There is scant reference to education as a stakeholder, no reference to involvement in providing mental health support, and limited linkages between education and mental health policies or legislation to support children and young people:

*“The general is that we keep stigmatising mental health… I repeat, we believe, not only in the Colombian education system but in general, it is stigmatised and people believe that working on your mental health does not involve prevention and promotion, but only medical care” (E005)*.

What came through clearly in the system architecture and how this supported young people to access care, was the siloed departmental working. The 2020 CONPES 3992 suggested that implementation would be through effective articulation of services between the different ‘areas of operation’ which were deemed to be the national and the territorial entities. Individuals with mental health problems were supposed to receive comprehensive care (*atención integral*) from the health system, as well as options to access services that guaranteed a dignified quality of life. Whilst the intent was clear and mirrored other policy objectives and principles, health systems predominantly target disorder rather than working across the mental health continuum (E007).

It was observable that accountability was held at the national level, with unspecified or unclear links to territorial counterparts. Budgeting or financial responsibility was predominantly articulated as operating at the macro level. Formal accountability and financial instruments were centralised. Conversely, many of the complex actions such as building rapport and service delivery through local populations were detailed as needing to take place at the meso (territory or regional) level and community engagement takes place through professionals providing services in communities (D14, D15, D24, D25, D26, D27).

This theme of over-centralisation appeared in the interview narratives, mirroring the observation from the documentary analysis whereby the national bodies were often accountable for priorities or policy goals, but with the work delegated to territories or communities. This over-centralisation was seen to be problematic because it created confusion centrally about who was accountable for what aspect of policy, and at the local level around implementation:

*“… many times, the remaining feeling is that their policies (…) can be defined at a very central level. And then, when these policies are to be implemented in such diverse regions, like the ones we have in the country, many times, we lack mechanisms or tools to achieve an adaptation that allows these policies to preserve the work lines they include and that differentially responds to the particularities of each of the territories (E006)”.*

Of note also, was the lack of integrated understanding about the role of NGOs in formal policy documents at national, territorial or regional strategy. This contrasted with the interview participants who highlighted both the important role of community, and the presence and role for NGOs in delivery of mental health care, and in local level partnerships. This was particularly important in rural and remote regions where there is little or poor rural mental health or health system infrastructure. The role for NGOs in the system architecture was seen to be in supporting ‘community tissue’ to enable mental health promotion:

*“With teachers, children and parents, they [NGO’s] are figuring out how to rebuild this community tissue that can promote mental health…I think they’re evidencing that there is an awareness that children need to have some development processes that can’t be interrupted, and that we have to protect these processes because, in the end, the mental health results are affected (E007)”.*

Views of service delivery, levels and responsibilities are further complicated by unclear definitions of ‘community’. Community was variously referred to but identified generally as being a key stakeholder in relation to care and services linked to geographical location (D14, D18, D26, D27). There was demonstrable lack of understanding of the impact of culture and ethnicity in relation to ‘community’. Interestingly, the WEF [1] principles include a specific one for *family engagement and support* but not an understanding outside this nuclear model of the significance of community. This contrast is significant, as it highlights how in conflict and post-conflict contexts differential approaches to who services are targeted towards, and how societies are organised to respond to mental health needs are not conceptualised similarly.

Macro level stakeholders engaged in discussion about the structural barriers to youth mental health service provision including: poor implementation; underdeveloped health systems in many areas; lack of resources (including staff); unequal distribution of resources; socioeconomic inequity (variable cost of treatment); and particular challenges with spatial differences relating to conflict affecting coverage and provision of services (E006).

Most documents examined in this research articulated clear barriers to implementation that can be divided into structural (implementation), and infrastructural (access) factors, including: disjointed/fragmented provision of services (D14); supply issues including human resources and medications (D14, D27); low availability of resources (D10, D14, D15, D18, D23, D27); poor information management systems (D14); problems with access and lack of investment (D14, D27); ignorance of services (D14); poor coordination of support care (D17, D18, D27); and a lack of talking therapy with only pharmacological intervention available (D14, D18, D25). Barriers provide useful insight into priorities for implementation, policy development and resourcing. One obvious tension was the lack of reflection or explicit analysis of the impact of delivering services in a conflict context. The understanding of conflict was confined to describing the impact on victims, making it difficult to develop population level policy and implementation that address goals of national and territorial integration.

A final pervasive implementation theme was a lack of specific action planning against the issues identified (D24, D26, D27) and the fragmented nature of action itself, frequently in silos (D2, D13, D16, D17, D18, D24, D27). Whilst there were territory/national agreements for some areas of policy, generally there was little or no evidence of joined-up priority setting or linking across sectors. This resulted in the uneven implementation issues evidenced by the quantitative analysis.

### The supply of health services and the currently understood demand

The analysis of the NMHS reveals that the prevalence of mental health problems in Colombia is relatively low, indeed, 4.5% [CI 3.4–5.6%] of individuals aged 12 to 17 years old had a mental health illness and 5.4% of young adults (18 to 24 years) [CI 4.5–6.9%]. In most cases, the prevalence of individual mental health illness was lower than one or two% depending on the age group and the mental health illness we were studying. For those self-reporting mental health illness for individuals aged 18 to 24 years, 1.2% referred to having a mental health episode in the last 12 months. In the case of adolescents, 3.4% reported having a mental episode and of this group 36.1% sought to receive mental health support.

We disaggregated the percentage of individuals who reported mental health services by region and found that in the case of the Pacific Region, 6.1% of the children aged 12 to 17 had any mental health illness in the last 12 months. In the case of individuals aged 18 to 24 years the highest prevalence of mental health illness was in the Pacific region, with 10.2% of the population in this age group reporting any type of mental health illness and the lowest in the Atlantic region (1.5%). Unfortunately, the sample size in most of the cases did not allow us to disaggregate or analyse in more detail the distribution of the prevalence of mental health illness in the country.

**Fig. 2:**
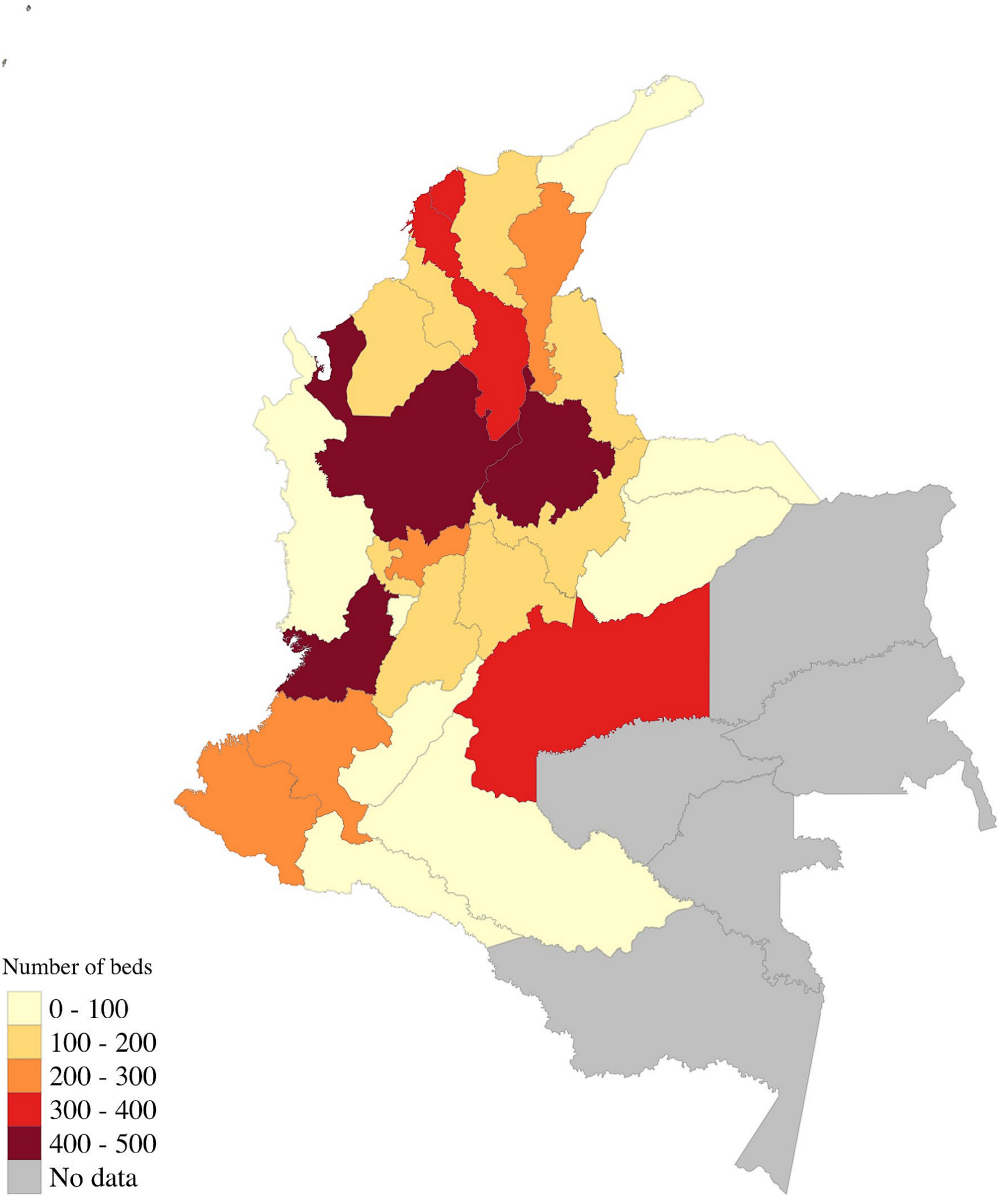
Distribution of Adult Mental Health beds in Colombia 2021

The analysis of REPS demonstrated an unequal distribution of mental health care services across the country with most services in populous regions or capital cities (see Fig. 2). Most services are located in urban areas and there are lower numbers of mental health professionals (psychiatric and psychologist) in rural and remote areas. In the case of institutions that provide psychiatric services, 1,968 institutions were identified that have outpatient services. Of those, 63% were offered by institutions providing healthcare services, 35% as individual providers and 2% as institutions with an objective different than healthcare services provision. Finally, 44% of the institutions are located in the four largest cities of the country (Bogota, Barranquilla, Cali and Medellin) and only 2% (41 services) are in the two poorest departments of the country (Choco (13) and La Guajira (28). It is important to highlight that of these institutions, none offers mental health services for children.

A similar pattern was found for psychological services. In 2021, 10,179 institutions or professionals were registered to provide psychological services. Of those, 61% were IPS, 24% were private individual providers and 15% were institutions whose main objective was not to provide healthcare services. There is unequal distribution of services, with 33% of psychologists based in the largest four cities of the country (Barranquilla, Bogota, Cali and Medellin) and only 3% in Choco and La Guajira. There are no specific services registered as only for children, despite us being aware that services are provided to this specific group.

The analysis of the percentage of individuals 12 to 25 that died by suicide according to the national mortality register revealed that 1.2% of individuals in this age group died by suicide in 2019, and it was the second cause of death with almost 10% of the population who died of violence dying because of this cause. We calculated a Pearson correlation to analyse the association between the number of mental health facilities and higher suicide rates in those areas, we found a negative and significant correlation of -0.45 (p-value 0.000). Triangulation of the REPS, NHMS and Suicide Rates (administrative records) data shows an association between the lower number of professionals and higher suicide rates in those areas.

### Impact of internal armed conflict on children and young people’s mental health needs and importance for future policy development

There is little recognition in policy of the impact of conflict on the mental health system in relation to the architecture and to the drivers of mental health needs. Our analyses identified three key impacts of the conflict on the mental health system and consequent service provision for young people: (1) Disruption to the mental health system; (2) Displacement of persons and impact on young people and families; and (3) Intergenerational impact of conflict.

From both the documentary policy, qualitative and quantitative analysis it was clear that conflict primarily impacted upon services by *disrupting* the mental health system (both the regionally and nationally delivered health services as well as any community or NGO provided services) to create unequal distribution of services and consequently impacting patterns of access to services:

*“One of the recurrent situations that we are starting to see again getting worse in the case of Colombia is the effect, the isolation which populations are subject to, and, in particular, additionally, the Colombian Pacific is experiencing it a lot. These isolations cause, well, absolutely every type of restriction, be it on goods and basic services, among them, food (E008)”.*

Interview participants suggested that access was especially disrupted to services and there were problems delivering services in rural areas (E004, E006, E008). In addition to the lack of basic services, problems with food, or accessing education in rural areas, it was clear that implementation of national policy or service infrastructure relating to mental health was poor and that non-pharmacological care was particularly sparse. This absence of state services paralleled understandings of how communities have developed ‘mechanisms’ to cope with poor mental health, but that these are often impacted or at times controlled by armed groups which further isolates rural individuals from the health system. Evident from the analysis of impact on the provision and delivery of mental health services, was the *impact of displacement* on young people and families. Displacement creates both emotional as well as practical constraints:

*“I mean, the armed conflict came in and affected my economic status, because I was banished from my lands, and this of course has a significant emotional impact on my roots, my identity, my belonging. If culturally I wasn’t allowed to bury my dead relatives, so I had to live and leave them thrown there, my mourning was affected, right? (E009)”.*

Young people were reported as being frequently internally displaced by conflict, and those not directly affected often chose to move out of their territories due to threats of violence or to avoid being recruited. There was also acknowledgement that poverty and socioeconomic pressure drove youth displacement:

*“Young people that are in armed conflict territories have lost interest in institutions and the school. A young person is male or female, over 12 or 13 years old, he or she is a person with high risk. So, if you look at the composition of the areas of armed conflict, where there is displacement all over, the composition of the population that moves, thus abandoning the territory, abandoning the possibility of a life project in their territory, is mainly of women and young people (E004)”.*

For those young people who were displaced, the stigma attached to relocation or reintegration post conflict was often cited as both a barrier to accessing support and a driver of poor mental health. Internal armed conflict had a dual function in shaping policy as it operates as both a determinant of poor mental health by increasing mental health problems (E002); and as a barrier to access through issues such as armed groups control of access to services within territories or by hindering mobility making it difficult for individuals to access services (E006). This was especially the case for children, adolescents and young people. Youth displacement significantly increases the risk of poor mental health:

*“Children, adolescents and young people are not cared for, and during armed conflicts this is even more difficult to happen…so, in general, there’s an exclusion of these populations who are also the most vulnerable ones in terms of mental health” (E002).*

The interviews also highlighted the role of increased levels of interpersonal and domestic violence as a result of the wider contextual violence, and participants described the fractured experiences of children and families within the Colombian context. This poses challenges for policymaking as the traditional family structures, links and supports have often been broken by displacement:

*Orphan children are completely different from displaced children or displaced family right? Or from children of family…whose members have been reported missing. So, you can’t homogenize the policy only based on monetary issues. (E001).*

The awareness of how conflict impacts relationships, family structure and children’s experiences, coupled with an understanding that the harm generated by conflict is insoluble through monetary based policy initiatives alone within communities, indicates a key priority for mental health policy-making in conflict settings.

When considering the impact on families a clear theme relating to the *intergenerational impact of conflict* emerged. Considering the concept of chronosystem (time) as articulated in ecological systems theory was helpful in deepening our understanding of the importance culturally, socially and practically of understanding the intergenerational effects and impact of conflict:

“*These fright mechanisms that armed actors used, what was their impact on people. Of course, there’s post-traumatic stress in some people who have repeatedly been victims of different armed actors. But also, there is a very strong collective impact nobody talks about. We experience it every day, and obviously communities feel it, but they don’t talk about it… and in relation to children and adolescents, the situation is even poorer…the cross-generational impacts we talked about are completely related to unsolved mental health situations that are transferred, transmitted to the following generations (E009)”.*

This was described by one participant describing a wife’s pain 20 years later upon losing the father of her 8 year-old child as being “pain encrusted” (E006). This encrusting of pain translated across generations. Developing a clearer understanding of this phenomenon has significant practical implications for service provision and for policy:

*“It has generational effects on children, so mental health problems can’t be treated in a residual way. I mean, they won’t be psychologically better as a consequence of care measures. What is required, then? A specific design to address this is needed. So, it’s not the same suffering for women as men, not the same when the person suffers sexual violence, recruitment, kidnapping; it’s not the same suffering if you’re the son or grandson. So, for example, these generational effects of the war are being analysed in Colombia. In Europe, World War II generational effects have been greatly analysed, but this not the case for Colombia… The effects on children of family…who experienced kidnapping are completely different from those experienced by parents, right? Although there is a damage experienced in the family (E001)”.*

The need to explore intergenerational conflict and the formation of youth identity and identification is important given the context in which young people are developing. The core developmental tasks of identity formation in adolescence and the disruption that conflict causes are poorly understood and for conflict and post-conflict contexts the impacts are extensive.

## Discussion

This article sought to chart the topography of mental healthcare service provision that exists for young people in Colombia. The mapping presents a complex picture within Colombia of limited formal mental health service provision largely located in major cities; the significant omission of connecting education providers to youth mental health policy; and a strong emphasis on the differential approach, focusing on delivery through communities as well as at the individual level. The nuance within these thematic areas is important– and will require further research to determine both the underlying causal reasons to support forging new paths for future provision of mental health services to young people in Colombia and countries with similar characteristics. The analysis presented in this paper identified impacts of armed conflict, effects from having a policy shaped by a differential approach, and implications for narrow policy reach and implementation.

### Impact of armed conflict

Our work showed that the provision of youth mental health services was seen as being significantly impacted by conflict, both in creating access issues in rural areas and in terms of the effects on individuals, families and communities. Ecological systems theory was helpful in understanding the function of the chronosystem (time) in relation to both intergenerational and transgenerational effects of conflict. Further research is needed to understand how these operate and evolve to understand impacts on communities. However, the importance culturally, socially, and practically, of understanding the intergenerational effects and impact of conflict remains unrecognised in policy or system development.

There was also explicit reference made within the documents to the superficiality of international or Western concepts of mental wellbeing for populations when operating in a conflict or post-conflict context, coupled with a stated need for evidence alongside political support and integration of mental health policy into broader national policy in order to drive change. Policy building within this geopolitical context, with dominant Western models of working that are unsuited to the situated reality for conflict contexts, is complex.

These nuanced understandings were especially important when thinking about the function of community as defined by policy. Community exists in policy and narrative terms as an explicit stakeholder that has an almost invisible role as an active agent of providing mental health support. Community was seen as a locus of support or an exclusionary space that was conditional on a complex range of circumstances each affected by personal history, family history, displacement history and conflict. To have a system predicated on community supports risks creating a conditional system and further marginalisation amongst those already marginalised. The function of community especially in relation to proximal processes and social connectedness– relational networks i.e., family, school, local area– is impacted by the dynamics of internal or armed conflict. There is a need to develop a better understanding of what ‘community’ is, and how it either supports or excludes populations and the ways in which this happens. An enhanced understanding will enable polices to more accurately target excluded groups but also provides information about how to strengthen existing supports.

### Effect of the differential approach

The contextual understanding of how the Colombian mental health system intends to provide support to young people in a conflict and post-conflict context developed through the documentary analysis. Analysis demonstrated how the differential approach shaped services resulting in targeted services in key areas of need, for example, victim support, but also resulting in a gap in provision for whole-population-level mental health support. Education settings generally, for example, are an important informal provider of mental health support to children and young people, but are not clearly articulated or included in mental health policy, or linked to implementation plans or activity.

### Narrow policy reach and implementation

In relation to policy reach and implementation, three key themes stand out: separate or siloed working between ministries and consequently sectors within territories (education, health, NGOs); weak policy implementation, particularly in rural areas or specific regions most affected by conflict; and inequity of access to, and subsequent engagement with, mental health services. In addition to paucity of services, each of these themes help provide partial indications as to why young people may be unable to access or make use of existing services.

Over-centralisation of policy and accountability has been accompanied by lack of co-ordination and limited integrated working across institutions or Ministries. Policy silos have further fragmented the support available and solidified the boundaries between those who have access and those who do not. Youth were specifically identified as missing from services that were seen to be either focused on children, or on adults as they fall in gaps between these two approaches.

### Limitations

We acknowledge that the analysis presented in this article has some limitations. Firstly, although we used the most recent and best available nationally representative data in Colombia, we recognise that some data sources are not updated and that the information might not reflect the current situation. Secondly, the recruitment of policy stakeholders was limited to people working in this specific area at the national (macro) level, which was intentional in the design. However, this resulted in only being able to sample a small number of policy officers working directly or tangentially on the mental health and wellbeing of young people, our sample was limited. The qualitative work was impacted additionally by the need to interview online during the Covid-19 pandemic.

## Conclusions

The research identified a series of key gaps that acted as barriers to implementation identified within policy. These included things such as the identification of missing groups, in particular indigenous groups, and some clear priority areas or categories of need identified but with unclear evidence regarding the reason for their selection. Linked to this there was a lack of understanding about the importance of adopting an intersectional approach. Existing policy demonstrated a lack of nuanced understanding about culture as a heterogenous entity, lacking sensitivity to colonial legacy for contemporary trauma when framing the understanding of the conflict itself.

When we map the evidence from Colombia on to the principles of the *Global Framework for Youth Mental Health* framework, we can see the following priorities in relation to youth needs are unmet: rapid, easy and affordable access; awareness, engagement and integration, early intervention, youth partnership, continuous improvement, and prevention. This clearly shows that the current iteration of the framework is not really addressing the key issues of post-conflict youth mental health and requires significant adaptation to improve effectiveness.

Not only does the context limit the effectiveness of the framework but our evidence also shows that there are significant spatial disparities between communities leading to regional inequity in the provision, and access to, services. Specifically, there is clear evidence that lack of youth mental health service providers was acutely worse in the poorest rural areas, which also had the highest suicide rates. The lack of data and small numbers of diseases and low prevalence echoed a picture where early intervention, detection and treatment is not taking place and mirrored the issues identified with access and provision. In short, there is an unequal distribution of mental health care services across the country with most services in populous regions or capital cities and low prevalence detected, which we view as a proxy indicator of poor levels of access to services. Given that many of these areas have also suffered from or are experiencing conflict, it is true to say that some of the most acute need within Colombia is not being met, mirroring broader concerns about service delivery and peacebuilding in conflict-affected areas.

### Future work

The analysis conducted identified a series of key gaps in knowledge that are important areas for future research. These included the lack of understanding of the territorial, regional and local interpretation and implementation policy and consequent provision of services. Whilst we know that there is inequity in relation to provision of services, we do not understand how this links to lived experience or people especially in rural and remote areas. We do not know in these areas what the demand or need for services amongst young people is.

The initial evidence gathered for this paper also indicates that the differential (victim focused) model adopted in Colombia excludes other groups, critically including ‘youth’, traditionally the group most associated with recruitment, which may have serious long-term effects on mental health. The armed conflict has naturally shaped the development of policy and services, suggesting the existing international framework published by the WEF [1] needs adapting to make it relevant, inclusive and appropriate for conflict contexts.

### Electronic supplementary material

Below is the link to the electronic supplementary material.

Supplementary Material 1

Supplementary Material 2

Supplementary Material 3

## Data Availability

The datasets used and/or analysed during the current study are available from the corresponding author on reasonable request.

## Declarations

### Authors’ information

This paper is part of an interdisciplinary research programme coproduced by a Colombian and a British, funded by Colombian and UK resources and led by joint PIs, J-PA in Colombia and PJ in the UK.
