# Supplementary material for: Macro level system mapping of the provision of mental health services to young people living in a conflict context in Colombia
Source: BMC Health Serv Res. 2024 Jan 25;24:138. doi: 10.1186/s12913-024-10602-2 (PMC10811930; doi:10.1186/s12913-024-10602-2)
Supplement: Supplementary file 1 — Supplementary Material 1 [file 12913_2024_10602_MOESM1_ESM.pdf]

**Appendix: Primary Source Documents**

| Primary Source | Documents |
|---|---|
| D1 | Law 100 |
| D2 | Law 387 |
| D3 | The 2358 Resolution |
| D4 | The Constitutional Act T-025 |
| D5 | The National Mental Health Policy |
| D6 | Law 122 |
| D7 | Law 157 |
| D8 | Law 1414 / Epilepsy Law |
| D9 | The T-045 Constitutional Act |
| D10 | Law 1448 & the Regulation 4633 |
| D11 | Law 1566 |
| D12 | The National Policy in the Field of Mental Health in Colombia |
| D13 | Law 1616 |
| D14 | The Resolution 1841 : The Ten-Year Public Health Plan (2012-2021) |
| D15 | The National Plan for Psychosocial Rehabilitation for Coexistence and Non-Repetition |
| D16 | The Mental Health National Plan |
| D17 | The Resolution 2626 |
| D18 | The Psychosocial Care and Integral Health Care for Victims Programme (PAPSIVI) |
| D19 | The 4634 Regulation |
| D20 | The 4635 Regulation |
| D21 | The T-422 Constitutional Act |
| D22 | The Mental Health National Policy |
| D23 | The Psychosocial Restoration National Plan |
| D24 | The 4886 Regulation |
| D25 | The 089 Regulation |

| | |
|---|---|
| D26 | The 2626 Resolution |
| D27 | The CONPES 3992 |
