# Supplementary material for: Macro level system mapping of the provision of mental health services to young people living in a conflict context in Colombia
Source: BMC Health Serv Res. 2024 Jan 25;24:138. doi: 10.1186/s12913-024-10602-2 (PMC10811930; doi:10.1186/s12913-024-10602-2)
Supplement: Supplementary file 2 — Supplementary Material 2 [file 12913_2024_10602_MOESM2_ESM.docx]

**-Topic guide for interviews with government actors in relation to mental health at the national level-**

The following describes the protocol which is used as a basis for semi-structured interviews with government actors at the national level who work on the issue of mental health either directly or indirectly through aligned policy areas, and who have been involved in the process of designing national-level policies or plans on mental health. The interview aims to identify the perceptions of government actors about mental health service provision and target groups for service provision within the country. It also aims to determine how children, adolescents and young people have been included in the different services and what the gaps and opportunities are that the country faces in the provision of services for this population, especially in Afro-Colombian and black communities in the Pacific Region. Finally, the interview aims to identify how the conflict has created changes in the service provision, particularly to this population. Each qualitative, oral interview will last approximately one hour. This protocol is used as a topic guide, which can be adapted and expanded depending on the specific government actor interviewed.

**Interview protocol:**

**Mental Health System in Colombia**

- How would you describe the mental health system in Colombia?
- What are the main characteristics of the mental health system in Colombia?
- What role do you feel community services play or should play in mental health care provision?
- How do you consider that ‘differential’ perspectives (including direct conflict experience as well as intersections of ethnicity, gender and/or disability) are addressed or included in the current national mental health policies and plans in Colombia?

**Mental Health System and children, adolescents and young people**

- To what extent and how do you consider that children, adolescents and young people are included in the mental health system provision in Colombia?
- How are the specific needs and rights of children, adolescents and young people are addressed by the mental health system and mental health policies in Colombia?
- Which stakeholders/actors bear the greatest responsibility for implementing actions to address the needs of children, adolescents and young people?
- Which services form part of the provision of mental health services for children, adolescents and young people?
- What are the barriers and facilitators for the provision of mental health services for children, adolescents and young people in Colombia?
- Do you consider that access to mental health services is equal or depends in any way on the group (conflict victims, gender or ethnic background) that the person belongs to?

**Armed Conflict and Mental Health**

✔ How do you consider that the armed conflict in Colombia has affected the design and implementation of the mental health services in the country?

✔ How has the conflict affected access to mental health services for children, adolescents and young people?

✔ How do you consider that ‘differential’ (i.e. direct conflict experience, ethnicity, gender and disability) perspectives are addressed or included in the current national mental health policies and plans in Colombia?

Possible discussion topics: Conflict experience (former combatants and victims), gender equity, disability rights, different ethnic groups (include specific policies or other relevant topics to the interview here).

Opportunity to follow up by email or in the interview with some additional questions.

The following questions can be used with people who are familiar with the legislation:

Legislation

✔ Which regulations, public policies or programmes in relation to mental health in Colombia are you familiar with?

✔ How do you consider that the regulations, public policies or programmes that you mentioned have been implemented in practice in the country?

✔ Could you give an example of a case in which a regulation, policy or program you mention has been applied?

✔ In the case you described, what do you consider the success factors and limitations in relation to the implementation of this specific regulation, program or policy?
