# Supplementary material for: Macro level system mapping of the provision of mental health services to young people living in a conflict context in Colombia
Source: BMC Health Serv Res. 2024 Jan 25;24:138. doi: 10.1186/s12913-024-10602-2 (PMC10811930; doi:10.1186/s12913-024-10602-2)
Supplement: Supplementary file 3 — Supplementary Material 3 [file 12913_2024_10602_MOESM3_ESM.docx]

**Appendix tables of supplementary information**

**Table A1: Primary Source Documents.**

| Code | Document | Date of Published |
| --- | --- | --- |
| D1 | Law 100 | 1993 |
| D2 | Law 387 | 1997 |
| D3 | The 2358 Resolution | 2006 |
| D4 | The Constitutional Act T-025 | 2004 |
| D5 | The National Mental Health Policy | 2018 |
| D6 | Law 1122 | 2007 |
| D7 | Regulation 157 | 2021 |
| D8 | Law 1414 / Epilepsy Law | 2010 |
| D9 | The T-045 Constitutional Act | 2010 |
| D10 | Law 1448 & the Regulation 4633 | 2011 |
| D11 | Law 1566 | 2012 |
| D12 | Resolution 2626: The National Policy in the Field of Mental Health in Colombia | 2019 |
| D13 | Law 1616 | 2013 |
| D14 | The Resolution 1841: The Ten-Year Public Health Plan (2012-2021) Publication | 2013 |
| D15 | The National Plan for Psychosocial Rehabilitation for Coexistence and Non-Repetition | 2013 |
| D16 | The Mental Health National Plan | 2014 |
| D17 | The Resolution 2626 | 2019 |
| D18 | The Psychosocial Care and Integral Health Care for Victims Programme (PAPSIVI) | 2011 |
| D19 | The 4634 Regulation | 2011 |
| D20 | The 4635 Regulation | 2011 |
| D21 | The T-422 Constitutional Act | 2004 |
| D22 | The Mental Health National Policy | 2018 |
| D23 | The Psychosocial Restoration National Plan | 2018 |
| D24 | The 4886 Regulation | 2018 |
| D25 | The 089 Regulation | 2019 |
| D26 | The 2626 Resolution | 2019 |
| D27 | The CONPES 3992 | 2020 |
